# Cortical Activation Changes during Repeated Laser Stimulation: A Magnetoencephalographic Study

**DOI:** 10.1371/journal.pone.0019744

**Published:** 2011-05-10

**Authors:** Andrej Stancak, Jamaan Alghamdi, Turo J. Nurmikko

**Affiliations:** 1 Department of Experimental Psychology, Institute of Psychology, Health, and Society, University of Liverpool, Liverpool, United Kingdom; 2 Third Faculty of Medicine, Charles University Prague, Prague, Czech Republic; 3 Department of Cellular and Molecular Biology, Institute of Translational Medicine, University of Liverpool, Liverpool, United Kingdom; 4 Unit of Neuroscience, Pain Research Institute, Institute of Aging and Chronic Disease, University of Liverpool, Liverpool, United Kingdom; University Medical Center Groningen UMCG, The Netherlands

## Abstract

Repeated warm laser stimuli produce a progressive increase of the sensation of warmth and heat and eventually that of a burning pain. The pain resulting from repetitive warm stimuli is mediated by summated C fibre responses. To shed more light on the cortical changes associated with pain during repeated subnoxious warm stimution, we analysed magnetoencephalographic (MEG) evoked fields in eleven subjects during application of repetitive warm laser stimuli to the dorsum of the right hand. One set of stimuli encompassed 10 laser pulses occurring at 2.5 s intervals. Parameters of laser stimulation were optimised to elicit a pleasant warm sensation upon a single stimulus with a rise of skin temperature after repeated stimulation not exceeding the threshold of C mechano-heat fibres. Subjects reported a progressive increase of the intensity of heat and burning pain during repeated laser stimulation in spite of only mild (4.8°C) increase of skin temperature from the first stimulus to the tenth stimulus. The mean reaction time, evaluated in six subjects, was 1.33 s, confirming involvement of C fibres. The neuromagnetic fields were modelled by five equivalent source dipoles located in the occipital cortex, cerebellum, posterior cingulate cortex, and left and right operculo-insular cortex. The only component showing statistically significant changes during repetitive laser stimulation was the late component of the contralateral operculo-insular source peaking at 1.05 s after stimulus onset. The amplitude increases of the late component of the contralateral operculo-insular source dipole correlated with the subjects' numerical ratings of warmth and pain. Results point to a pivotal role of the contralateral operculo-insular region in processing of C-fibre mediated pain during repeated subnoxious laser stimulation.

## Introduction

Pain resulting from stimulation of unmyelinated C fibres dominates various types of acute and chronic pain. Therefore, understanding of cortical processing of C-fibre mediated pain is of special importance as it may identify potential targets in neurostimulation or pharmacological therapy of chronic pain.

Repeated subnoxious or mildly noxious thermal or mechanical stimulation at frequency greater than 0.3 Hz leads to progressive increase of sensations and eventually to burning pain. This phenomenon is known as temporal summation of pain (TSP) [1]. TSP appears to induce comparative long-lasting changes in excitability manifesting in bursts of pain occurring spontaneously during the course of a TSP experiment [2] and in enlargement of the receptive fields originally stimulated to produce TSP [3]. Common chronic pain syndromes such as fibromyalgia [2], [4], [5], spinal cord injury [6], complex regional pain syndrome [7], or temporomandibular syndrome [8] are associated with an elevated slope of TSP.

Pain evolving during repeated subnoxious or mildly noxious stimulation involves changes in neuronal excitability in the spinal cord. Converging evidence suggests that TSP is mediated by unmyelinated C fibres and wide-dynamic range neurons in the dorsal horn [9], [10], [11]. However, the firing patterns of lamina I second order nociceptive neurons also showed a temporal profile of summation during repeated heat stimulation [12].

In humans, cerebral changes occurring during TSP were investigated using fMRI [13], [14], [15] and EEG [16]. Staud et al. [13], [15] applied contact heat stimuli to analyse effects of repeated heat stimuli on brain activation and reported stronger activations in a number of brain regions after the last of six heat stimuli. However, prolonged contact heat stimulation leads to accumulation of heat under the thermode and therefore, the resulting activation maps are likely to be representative of a compound effect of all six stimuli occurring in one block. The comparatively low temporal resultion of fMRI does not offer the possibility to analyse the cortical responses to each repeated stimulus, and their correlation with subjective reports.

The purpose of the present study was to identify cortical regions that during repetitive warm stimulation of the skin demonstrate gradual increases of activity in parallel with perceived intensity of sensation of heat and pain in healthy subjects. In Experiment 1, we chose to apply low-energy laser stimuli that selectively activated C fibres and produced a small post-stimulus increase of skin temperature not exceding the thresholds of cutaneous mechano-heat receptors even after 10 repeated stimuli. To evaluate the frequency specificity of repeated laser stimulation on pain perception, laser stimuli were applied with frequency of 0.4 Hz, known to induce pain during temporal summation experiments, and with low frequency of 0.17 Hz that was shown not to induce pain during repeated thermal stimulation [13], [17]. Whole-head magnetoencephalographic recordings offering a temporal resolution on the scale of milliseconds was used to evaluate the temporal activation profiles in different brain regions during repetitive laser stimulation. The changes of skin temperature during repeated laser stimulation were analysed in Experiment 2. To demonstrate the specificity of laser stimulation, reaction times to laser stimuli were analysed in Experiment 3.

## Results

### Experiment 1

#### Perceptual changes associated with temporal summation of pain

**Figure 1:**
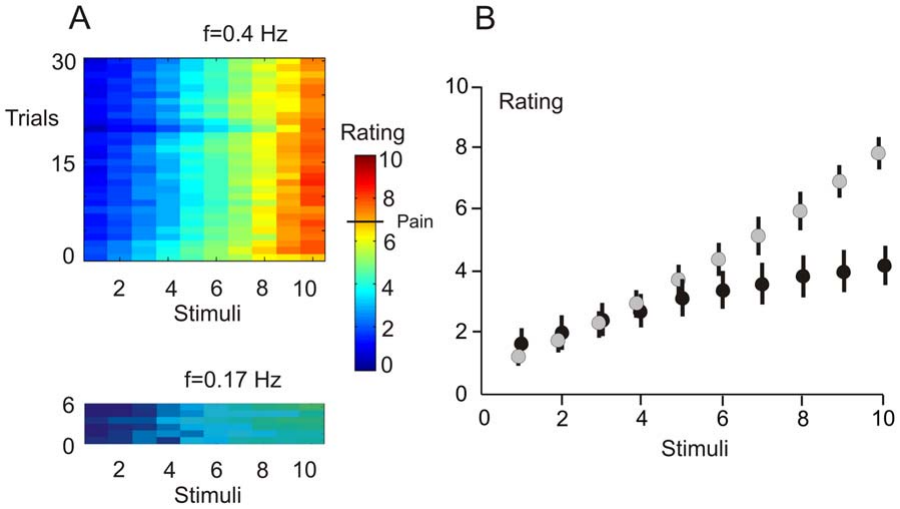
Perceptual wind-up at f = 0.4 Hz and f = 0.17 Hz. A. Panels depicting the stimulus-trial colour-coded plots of numerical ratings for 30 trials run with f = 0.4 Hz (upper panel) and with f = 0.17 Hz (lower panel). Rating values equal to or greater than 7 correspond to pain sensations. B. Graphical presentation of intensity of preceipts plotted against the incremental number of applied stimuli. The mean values and standard error bars of numerical ratings during blocks with f = 0.4 Hz (grey circles) and f = 0.17 Hz (black circles) are shown.

All subjects reported a pleasant warm sensation upon a single laser stimulus. However, repeated stimulation at f = 0.4 Hz produced an increasingly intense sensation of warmth, then heat and finally a burning pain upon stimuli 9 or 10. Stimulation at low frequency f = 0.17 Hz produced only a sensation of warmth not exceeding the scores of 4 or 5 on the numerical rating scale. Figure 1A shows the stimulus-trial plots of numerical ratings under f = 0.4 Hz and f = 0.17 Hz. Figure 1B illustrates the mean ratings and standard errors of the mean for each of ten laser stimuli. Two-way ANOVA for repeated measures (10 laser stimuli, f = 0.4 Hz vs. f = 0.17 Hz) showed a statistically significant main effect of stimulus repetition (F(9,90) = 92.5, P = 0.00001, Greenhouse-Geisser ε = 0.173), and a statistically significant interaction between repeated stimuli and frequency of stimulation (F(9,90) = 39.9, P = 0.00001, Greenhouse-Geisser ε = 0.203). The interaction was due to the greater perceived intensity of heat or pain sensations with the stimulation frequency of f = 0.4 Hz than f = 0.17 Hz after stimuli 7, 8, 9, and 10 (paired Student t-test, P<0.01) but not after stimuli 1–6 (P>0.05). The stimulated region of the skin was intact at the end of experiment. Some subjects showed a pink-coloured spot on the stimulated area of the skin which, however, disappeared within 5–10 min, suggesting a vasomotor origin.

Only four out of eleven subjects pressed the escape button to avoid another laser stimulus in some of the trials. This occurred in 11 trials after the 8th stimulus and in 44 trials after the 9th stimulus. Since the number of stimuli lost due to the subjects' interceptions was comparatively small (55 out of 660 stimuli, 8.3%), no special analysis was performed in regard to the trials followed by movement. However, as finger movements [18] and muscle contractions [19] attenuate pain-related cortical responses, all responses acquired after such movement were discarded from the analysis.

Data showed that our stimulation protocol involving repeated laser stimuli produced a temporal summation of warmth both at f = 0.4 Hz and f = 0.17 Hz. In accordance with previous studies in humans [13], [17], the temporal summation leading to pain was observed only at the frequency f = 0.4 Hz.

#### Source model

MEG waveforms, related to laser stimuli and to simultaneous changes in the visual field due to presentation of the stimulus order number, trials were averaged across trials and subjects and filtered from 0.3 Hz to 7 Hz. These filter settings have been chosen after testing impacts of different filter limits on source dipole models. As only 30 laser stimuli were administered for each stimulus order, the averaged MEG fields were modulated by spontaneous cortical oscillations >10 Hz. These phase-unlocked spontaneous oscillations contributed to the noise, and the source dipole models attempted to model this high-frequency noise. Setting the high-frequency limit to 7 Hz allowed us to model the slow, low amplitude MEG fields occurring about 1 s after stimulus onset. The mean sensor locations were also computed and used to model the grand average MEG waveforms by a set of equivalent source dipoles.

**Figure 2:**
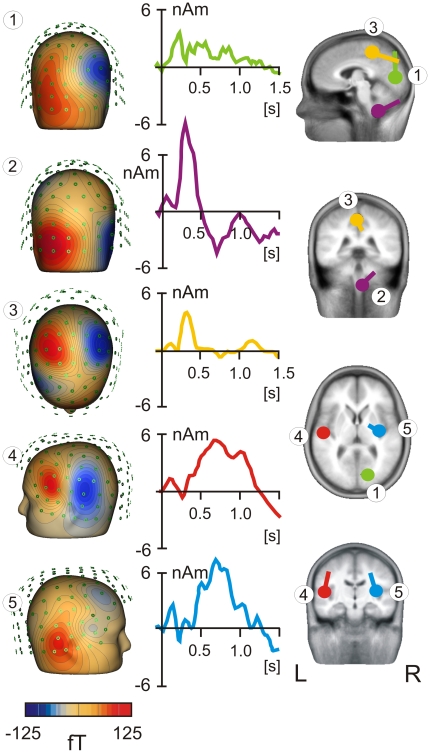
Source dipole model. Grand average evoked fields were modelled by five equivalent source dipoles. The isopotential flux maps for each source, numbered from 1 to 5, are shown in the left column. The source waveforms are in the middle column. Tha locations of all sources are shown in four slices in the right column. L = left, R = right.

The source locations, isopotential field patterns, and grand average source waveforms of each source are shown in Figure 2. The first source dipole was fitted in the occipital cortex (approximate Talairach coordinates: x = 17 mm, y = −78 mm, z = 7 mm; Brodmann area 17), and accounted for the early component peaking at 215±76 ms (mean ± SD of peak latencies obtained in 11 individual averaged source waveforms comprising all 10 laser stimuli). The second dipole, located in cerebellum, explained the topographic pattern occurring in the right suboccipital region. The peak latency of the cerebellar dipole was 290±83 ms. The third source dipole, peaking at 414±156 ms was found in the posterior cingulate cortex (Talairach coordinates: x = −3 mm, y = −37 mm, z = 47 mm; Brodmann area 31). Finally, there were two source dipoles explaining the magnetic fields occurring over the left and right fronto-temporal regions. These topographic patterns of magnetic fields were explained by two dipoles located in the left and right operculo-insular regions. The left source was situated in the Sylvian fissure corresponding to the secondary somatosensory cortex (SII, Talairach coordinates: x = −50 mm, y = −16 mm, z = 12 mm). The source waveform showed one peak at 622±123 ms and another at 1047±179 ms. The right-hemisphere source was located in the right operculo-insular cortex (Talairach coordinates: x = 33 mm, y = −15 mm, z = 14 mm). The maximum source activity in the right operculo-insular cortex occurred at 729±156 ms.

We have specifically analysed whether the source activity in the left operculo-insular region would be better modelled using one dipole or two source dipoles located in the posterior insula and in the Sylvian fissure. However, the six-dipole solution involving two source dipoles in the left operculo-insular cortex was not optimal as one of the two sources explained only 1% of residual variance and had a flat source waveform. To validate further modeling of MEG fields in the left fronto-temporal region, the source probe scan method, implemented in BESA program, was applied. The source probe scan maps represent the distribution of Z values that are based on signal-to-noise estimations of the probe source waveform and allow evaluation of the number of source dipoles in a specific cortical region. The source probe scan map showed no consistent changes in the shape of source activity over 10 repeated laser stimuli leading us to accept the five-dipole source model with one source dipole modeling the cortical activity in the left operculo-insular cortex.

This five-dipole model proved to be stable both in grand average data and in individual subjects and conditions. To evaluate the source dipole moments in a particular stimulus and subject, the locations of the five source dipoles were kept fixed and the source dipole orientations were re-fitted in each subject.

#### MEG changes during repeated laser stimulation

**Figure 3:**
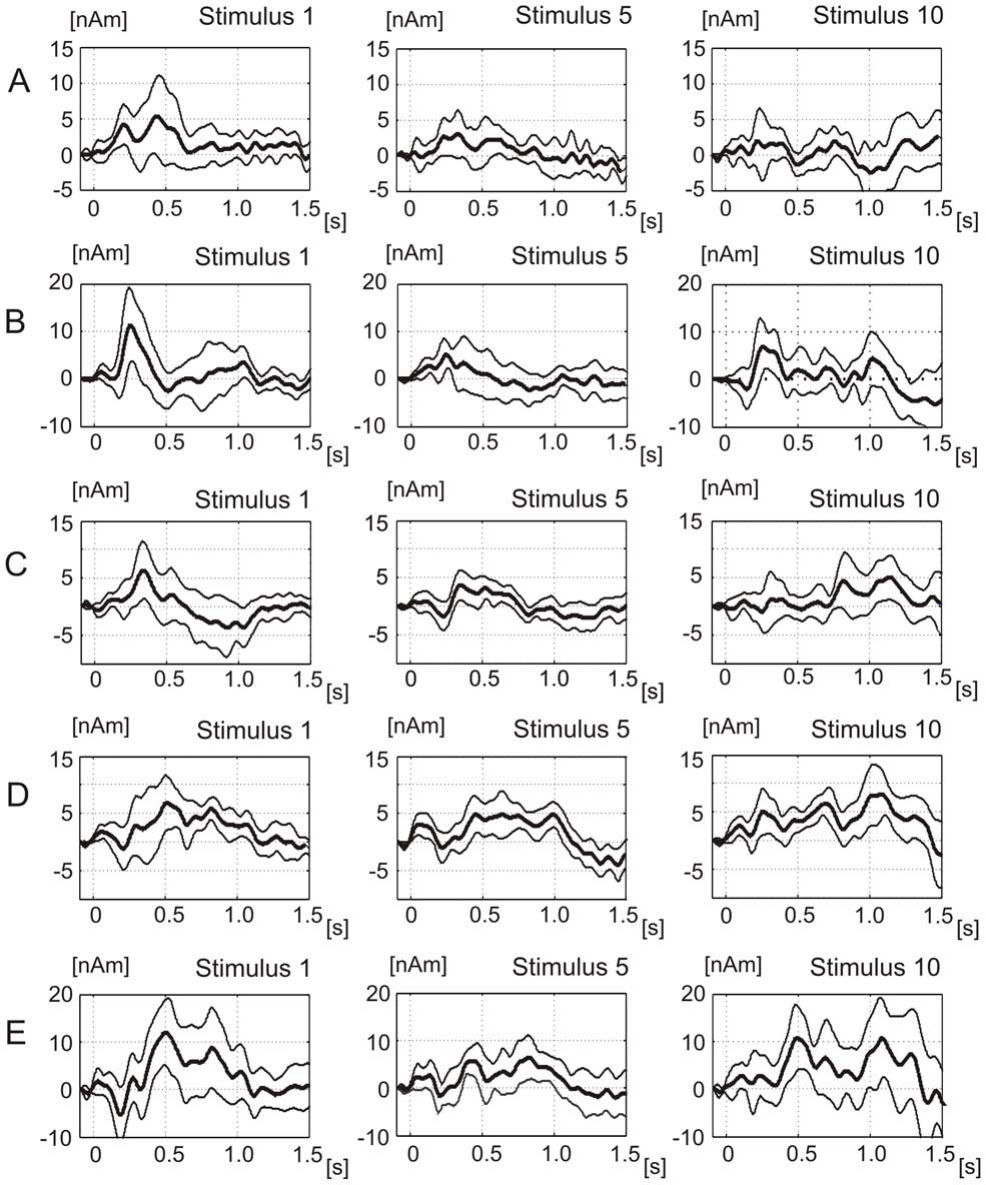
Source waveforms in five source dipoles during stimulus 1, 5 and 10. The plots show the average (N = 11) source waveforms and the 95% confidence intervals for three of ten laser stimuli. A. The occipital cortex source (dipole 1 in Figure 2). B. The cerebellar source dipole 2. C. The posterior cingulate source dipole 3. D. The left operculo-insular source dipole 4. E. The right operculo-insular source dipole 5.

Figures 3A–3E show the grand average source waveforms and the 95% confidence lines of each of five source dipoles during the first, fifth, and tenth laser stimulus. The occipital and posterior cingulate source showed a comparatively strong activity during the first laser stimulus, and a decay of source activity during subsequent stimuli, illustrated for stimuli 1, 5 and 10 in Figures 3A–3E. The cerebellar source (Figure 3B) and the right operculo-insular cortex showed an U-shaped profile of amplitude changes. The left frontoparietal-opercular source showed progressive increases of amplitudes of the early component around 600 ms and especially of the late component around 1100 ms. The source dipole moments of the late components of the operculo-insular sources were comparatively small but reliably exceeded the baseline noise level (Figures 3D–3E). These small source dipole amplitudes are attributable to the very low intensity of laser stimulation capable of evoking only a mild warming sensation upon a single presentation. The mean values of peak latencies and peak source amplitudes in stimuli 1 and 10 are listed in Table 1.

**Table 1:** Mean values (± SEM) of peak latency and peak source dipole amplitude during stimuli 1 and 10.

	Peak latency [ms]				Peak amplitude [nAm]			
	Stimulus 1	Stimulus 10	t(10)	P	Stimulus 1	Stimulus 10	t(10)	P
Source 1	232±15	234±21	0.11	0.91	7.7±2.2	5.9±2.1	0.66	0.52
Source 2	282±13	304±18	0.99	0.34	8.8±2.0	8.0±1.6	0.32	0.76
Source 3	412±30	432±31	0.48	0.65	8.5±2.4	4.8±1.3	1.57	0.15
Source 4 (620 ms)	618±40.0	632±36	0.39	0.70	8.6±1.0	10.3±1.9	0.74	0.47
Source 4 (1050 ms)	976±31	1048±16	1.89	0.09	6.4±4.8	11.8±1.4	2.9	0.02
Source 5	643±40	693±34	1.0	0.32	19.1±3.9	17.2±2.6	0.41	0.67

Statistical evaluations of the differences in mean values, based on the Student's t-test for paired observations, are also shown.

**Figure 4:**
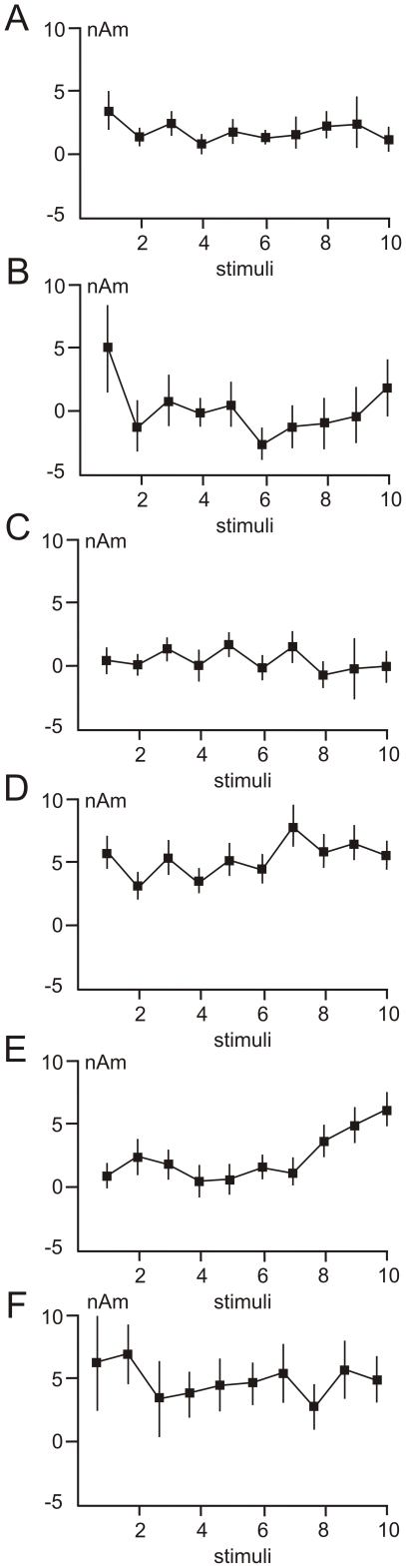
Mean values and standard errors of the mean of source dipole components during repeated laser stimulation. A. The occipital source dipole (labelled 1 in Figure 2). B. The cerebellar source dipole (labelled 2 in Figure 2). C. The posterior cingulate source (labelled 3 in Figure 2). D. The early component (t = 670 ms) of the left operculo-insular source dipole (labelled 4 in Figure 2). E. The late component (t = 1047 ms) of the left operculo-insular source dipole (labelled 4 in Figure 2). F. The right operculo-insular source dipole (labelled 2 in Figure 2).

One-way ANOVA for repeated measures was performed with source dipole strengths for each of the five source dipoles as dependent variables and ten successive laser stimuli as the independent variable. The source amplitudes were computed as average source dipole moment in epochs centred at the grand average maximum. The widths of the epochs were identical for each condition and subject but differed in five dipoles as the epoch durations were equal to the standard deviations of the peak latencies. In the contralateral operculo-insular source, the source strengths were evaluated in each of the two peaks. The mean values and standard errors of the mean of source dipole components in ten stimuli are shown in Figures 4A–4E.

The only variable showing statistically significant changes of source strengths over repeated laser stimuli was the late component of the left (contralateral) operculo-insular source dipole (F(9,90) = 2.9, P = 0.035, Greenhouse-Geisser ε = 0.432). This effect was due to greater source amplitude in stimulus 10 (two-tailed Student's t-test for paired observations, t(10) = 3.82, P = 0.003), and stimulus 9, t(10) = 2.9, P = 0.016) compared to stimulus 1 (Figure 4D). The statistically significant amplitude increase of the 1050 ms component in stimulus 10 compared to stimulus 1 was also confirmed using peak amplitude data (Table 1).

**Figure 5:**
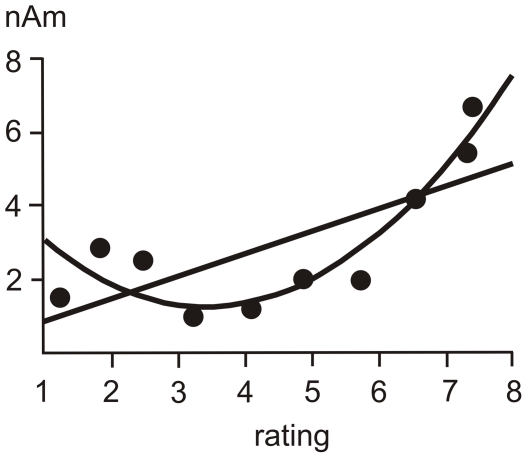
The linear and quadratic regression fits of the association between numerical rating values and the amplitude of the late component of the left operculo-insular source dipole. Data represent grand average values of numerical ratings and source dipole moments of eleven subjects.

Figure 5 shows the scatter plots of amplitudes of the late operculo-insular component and numerical ratings in grand average data. The quadratic function fit accounted for as much as 86% of variance, and the regression model in the form y = 5.22−2.57×x+0.44×x^2^ proved to be statistically significant (F(3,7) = 59.9, P<0.0001; Pearson's correlation coefficient r (10) = 0.93, P<0.0001). The quadratic relationship between subjective reports and source dipole moments in the left operculo-insular cortex suggests that only strong heat and pain sensations are featured in the contralateral operculo-insular cortex.

### Experiment 2

**Figure 6:**
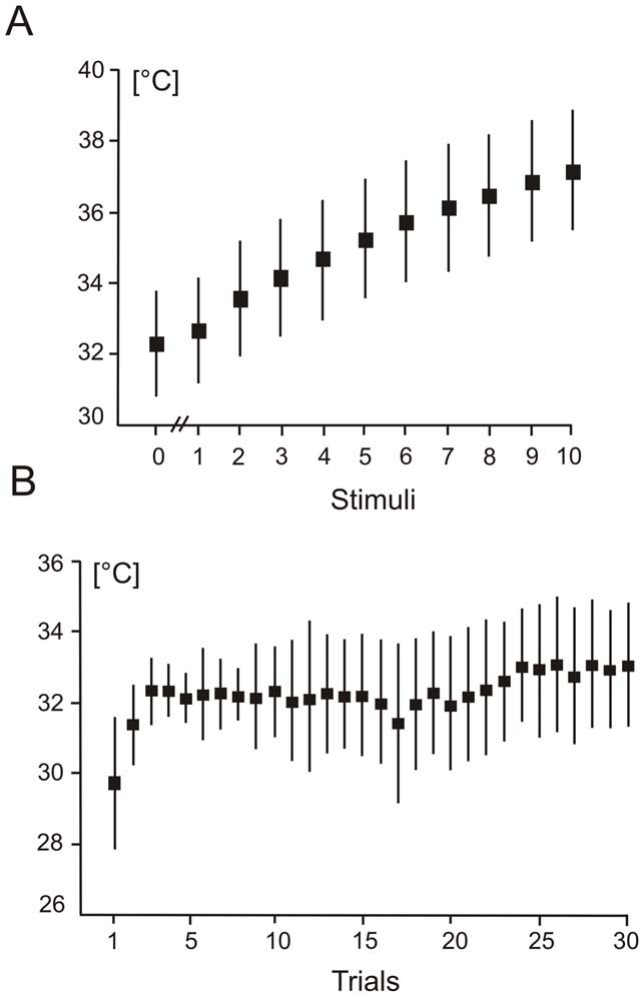
Temperature changes during repeated laser stimulation. A. Mean temperature values preceding the first laser stimulus (labelelled “0”) and following each of 10 laser stimuli. B. Trial-by-trial baseline temperature values. The vertical vertical bars represent the standard deviations.

Figure 6A shows the mean temperature changes prior to the first laser stimulus and after each of 10 laser stimuli. The skin temperature gradually increased from the mean value of 32.3±1.4°C at baseline to 37.1±1.7°C that was recorded after the last laser stimulus. The increase of temperature from stimulus 1 to stimulus 10 was statistically significant according to one-way repeated measures ANOVA (F(9,36) = 351.5, P<0.0001, Greenhouse Geisser ε = 0.111).

Figure 6B illustrates the changes of baseline skin temperature evaluated from skin temperature values recored prior to the first laser stimulus in every of 30 trials. The basal skin temperature increased from trial 1 (29.7±1.8°C) to trial 3 (32.3±0.7°C), and remained at a relatively constant level up to the trial 24 to gradually increase to 33.1±1.8°C upon trial 30. Thus, after the initial increase the mean basal temperature fluctuated in a relatively narrow range between 32 and 33°C.

### Experiment 3

The overall mean reaction time was 1.33±0.27 s (mean ± SD). Reaction time was slightly longer in the first half of stimuli, during which subjects perceived mild warming sensations, compared to the second half of stimuli. However, the changes of the mean reaction time over 10 laser stimuli were not statistically significant according to one-way ANOVA for repeated measures (F(9,36) = 2.52, P = 0.158, Greenhouse Geisser ε = 0.177). The reaction time data supported the view that the laser-induced warm and heat pain sensations were mediated by the slow-conducting C fibres.

## Discussion

Repeated innocuous warm stimuli presented at a frequency of 0.4 Hz induced a progressive increase of heat and pain sensations. The low-energy laser stimulation used in this study presumably activated non-nociceptive C fibres because the maximal increase of skin temperature was below the range of cutaneous C mechano-heat nociceptors [20], [21], [22]. As the maximum skin temperature, reached after the last of 10 consecutive laser stimuli, was not sufficient to produce primary hyperalgesia, as seen in some nociceptive C fibres after prolonged or repeated noxious heat stimulation [23], [24], we assume that the pain evolving during repeated laser stimulation resulted from temporal summation of C-fibre responses occurring within the central nervous system. The return of skin temperature to pre-stimulus level following a high-energy Nd-YAP laser stimulus may take one minute [25]. In our experiment, the basal skin temperature showed an initial increase of basal temperature during the first three trials and a comparatively stable baseline of 32–33°C during the rest of trials. These basal temperature values are substantially below the sensitivity range of the mechano-heat C-fibre nociceptors [22]. Therefore, we assume that the pain evolving during repeated laser stimulation in the present study involved the mechanism of temporal summation of pain as the somatosensory input was not sufficient to stimulate or sensitise the nociceptive C fibres.

The novel finding is that the neuromagnetic fields generated in the contralateral operculo-insular cortex showed progressive increases paralleling the subjects' perceived intensity of the sensation of heat and pain, linking this region to cerebral processing of C-fibre mediated pain and temporal summation of pain. The time course of activation in the the left operculo-insular was similar to the time courses of the overall MEG activity, as well as activities in the contralateral opercular cortex and the rostral anterior cingulate cortex during selective C-fibre laser stimulation [26]. The mean peak latency of the contralateral operculo-insular source showing an amplitude increase during repeated laser stimulation was 1.05 s which fits with the latency of the cortical oscillatory responses seen during innocuous warming of the hand [27], [28]. The latency of the early operculo-insular component (622 ms) is compatible with the latency of warm-related evoked potentials peaking at about 500 ms [29], [30]. Thus, the cortical activation changes in the contralateral operculo-insular cortex corresponded to the latency of second pain, reported to develop during repeated heat stimulation [1], [17]. The burning pain perceived by the subjects during stimuli 9 and 10 is likely to be attributable to C fibre stimulation [31], [32] as laser stimulation of Aδ fibres gives a sharp, pricking pain [33], [34]. The overall mean reaction time during repeated laser stimulation was at a comparatively stable level of 1.33±0.27 s. This comparatively long latency also involves the central processing time estimated to be about 0.27 s for noxious laser stimuli [35], and it is consistent with the latency of the late operculo-insular source component. Therefore, we conclude that the activation changes in the contralateral operculo-insular cortex were related to the C fibre volleys.

The source dipole manifesting statistically significant amplitude changes during repeated laser stimulation was located at the intersection of the parietal operculum, corresponding to the secondary somatosensory cortex, and the posterior insula. We assume that this source picked up activity from both cortical regions because the generator area of MEG sources encompasses a cortical tissue sized several cm^2^ [36], and it is therefore likely that a source located at the boundary of SII and posterior insula was influenced by both cortical structures. Involvement of the dorsal posterior insula and SII in the temporal summation of pain accords with previous brain imaging studies frequently reporting a co-activation of SII and posterior insula [37], [38]. Recently, Dum et al. [39] indentified the cortical projections of the spinothalamic tract in monkeys. The posterior insula and the adjacent SII were two major target zones accounting for 41% and 29% of spinothalamic projections, respectively. Thus, we assume that both the SII and the posterior insula contributed to the source dipole manifesting a temporal summation during repeated laser stimulation.

The present data emphasises the role of the operculo-insular cortex in processsing of C-fibre input during repeated laser stimulation. Direct electrical stimulation of posterior insula [40] and SII [41] was shown to produce painful sensations that have not been elicited during stimulation of any other cortical regions. Interestingly, burning pain sensations, reported by the subjects in our study, were only evoked during stimulation of posterior insula [40] but not during stimulation of SII [41].

It has been suggested that lamina I neurons would project primarily to the posterior insula via thalamic ventromedial posterior nucleus, whilst lamina V neurons would project to SII [42] possibly via the ventroposterior inferior nucleus [43]. Our data, showing one contralateral operculo-insular source dipole explaining the activation changes during each of ten repeated laser stimuli, suggests that both cortical regions and presumably both labelled lines were activated simultaneously. However, simultaneous recruitment of lamina I and lamina V neurons during repeated warm stimulation does not rule out functional differences of lamina I and lamina V projections with non-specific priming of the brain stem and premotor and motor cortical regions by lamina V neurons and nociceptive-specific activation of dorsal posterior insula by lamina I neurons [44]. Frot et al. [45] showed, using intracerebral recordings, simultaneous activation of posterior insula and SII during non-noxious and noxious laser stimulation with SII responding to gradual increases of temperature and insula responding only to noxious stimuli, which suggests different roles for the two co-activated areas.

### Pain specifity of laser-induced cortical responses

Our data contrasts recent EEG studies casting doubts on the specificity of the cortical responses following single or repeated laser stimuli activating the cutaneous Aδ fibers [46], [47]. Mouraux and Iannetti [46] argued that the laser-evoked potentials reflected modality non-specific cortical activation that can be evoked equally by noxious laser stimulus and innocuous somatosensory or visual stimuli. Iannetti et al. [47] reported a decline of laser-evoked potentials over the sequence of three laser stimuli, and showed that stimulus saliency rather than stimulus intensity accounts for the amplitude variations of the laser-evoked potentials. Indeed, the present data showed a certain mild decline of source activities in several sources including the left operculo-insular cortex that would fit the adaptation of the cortical responses seen in previous study [47]. However, the present study also shows that increasing the number of C-fibre mediated stimuli unmasks the pain-specific cortical activation. Our study shows that the pain-specific components elicited by laser stimulation originate in the contralateral operculo-insular cortex and show a consistent non-linear relationship with the intensity of the sensations of heat and pain. The C-fibre related nociceptive activation in the operculo-insular cortex accords with both recent neuroanatomical evidence about projections of spinothalamic neurons [48] and neurostimulation studies in humans pointing to operculo-insular cortex as the sole cortical region capable of producing pain sensations during direct electrical stimulation [40], [41].

It should be noted that the evoked magnetic fields were also generated in the visual cortex, posterior cingulate cortex and cerebellum prior fields originating in the operculo-insular cortex. The shortest latency had the source located in the visual cortex, which was related to the change in visual field due to presentation of a new stimulus order number. The responses in the posterior cingulate cortex are best explained by anticipation and preparation for a motor response such as withdrawal or button-pressing [49], [50]. The fact that none of these regions showed any significant change in source activity during repeated laser stimulation and that their peak latencies were relatively short strongly suggests that the activations were independent of sensory processing of C-fibre input.

### Limitations of the study

As MEG is sensitive only to tangential sources located predominantly in the fissural cortex [51], there is a possibility that a source with an exclusive radial orientation, not seen in MEG, might also show progressive amplitude increases associated with the temporal summation of pain. Typically, laser-evoked EEG potentials related to Aδ type laser stimuli show a prominent N2 component originating in the anterior cingulate cortex [33], [52], [53], [54], whereas reports on MEG sources in the anterior cingulate cortex are sparse. This issue will be addressed in a future study.

### Conclusion

To conclude, the long-latency evoked field component of the source dipole located in the contralateral operculo-insular cortex shows a progressive increase of activation during repeated laser stimulation that paralells, in a non-linear fashion, the increase in intensity of reported sensations. This suggests that the operculo-insular cortex represents the key cortical structure involved in the process whereby innocuous peripheral thermal stimuli become intensely painful. While this study does not answer the question of where in the neural pathways such a transformation takes place, identification of a specific cerebral substrate for the phenomenon will likely have wider applications. The operculo-insular region has been shown to be associated with encoding the intensity of noxious stimuli [55] and is also implicated in chronic pain [56]. It is of interest that patients with lesions localised to this region not only show abnormalities of thermal sensation but also mechanical and cold allodynia, and frequently report burning pain [56], [57]. As allodynia, temporal summation is one of the hallmark signs of neuropathic pain and remains a major clinical challenge. Our findings suggest that future studies are warranted to explore the potential of this region to evaluate if it can serve as a target for new therapeutic pain-relieving interventions.

## Materials and Methods

### Experiment 1

#### Subjects and Procedure

Eleven healthy subjects (28.1±6.5 years, mean ± SD) took part in the study after giving written consent. The study was approved by the Ethical Committee of the University of Liverpool.

Subjects received a detailed explanation of the procedure prior to being placed on the MEG scanner table. Five head coils were mounted on the head and the the head shape was measured using Fastrack (Polhemus Navigation, Colchester, USA). Laser stimuli were produced using a Nd:YAP laser stimulator Stimul 1340 (El.En. S.p.A., Firenze, Italy) operating with 1340 nm wavelength. The stylus of the laser stimulator was mounted on a wooden stand and an 8 cm distance between stylus and the dorsum of the right hand was maintained. In line with previous studies [30], [58] and preliminary pilot experiments, the parameters of laser stimulation were optimised for stimulation of warm C fibres known to be located supeficially in the skin, and have small receptive fields [59], [60]. The diameter of the laser beam was 14 mm, pulse duration was 10 ms, and energy was 5.5 J. The energy density applied on each stimulus was 3.54 J/cm^2^. In every subject, single laser stimuli of this type produced a mild warming sensation. The energy used was sufficiently low enough not to pose a danger of a burn or an alteration in skin pigmentation. Subjects had an emergency press-button switch in their left hand which they could use to terminate the set of stimuli at any time. They were instructed to press the button if they felt that the next stimulus might cause intolerable pain. Each set of stimuli comprised 10 laser stimuli or less if the escape button was used. The order number of the stimulus was projected on the mirror screen placed about 1 m above the scanner table. The order numbers were shown to facilitate the reporting by the subjects of the numerical ratings of warmth and pain. Five seconds after the last stimulus of each set, up to ten vertical yellow bars each having 10 divisions were shown on a blue background. Each scale box ranged from 0 (no sensation) to 10 (worst possible pain) with 7 representing the detection threshold for pain. The press-button switch the subjects held in their left hand was used for intensity rating. When the button was continuously pressed the boxes were incrementally illuminated at a rate of one box per 0.5 s. The participant would release the button as soon as the correct intensity box level was highlighted; this was taken as the intensity reading. At this stage, the cursor moved to the next vertical box scale to be used in the rating of the next set of stimuli. The rating scales were replaced by a fixation cross after the subject had scored the last stimulus of each set for 15-s resting interval. Subjects were trained in the use of numerical rating scales before the experiment.

The experiment was organised into two blocks of stimuli. In the first block, six sets of 10 laser stimuli with an interstimulus interval of 6 s (f = 0.17 Hz) were acquired. The purpose of this block was to demonstrate the absence of temporal summation of pain if the stimulation frequency was less than 0.3 Hz. The first block of the experiment lasted about 10 min. In the second block, thirty sets of ten laser stimuli were applied with an inter-stimulus interval of 2.5 s (f = 0.4 Hz). MEG data from the second block of experiments, offering sufficient number of stimuli, were analysed. The duration of the second block of the experiment was about 35 min. The inter-trial intervals in the second block of experiments were 70–75 s.

#### Recordings

Whole-head MEG was recorded using 148 axial magnetometers encased in a plastic helmet (Magnes 2400, 4D Neuroimaging, San Diego, USA). The bandpass filter was 0–200 Hz, and the sampling rate was 640.78 Hz. Vertical electrooculogram was recorded using silver electrodes filled with conductive gel and placed above and below the right eye. Electrocardiogram was recorded by two silver cup electrodes placed on the left and right shoulder. In five subjects, high-resolution MR images of the head were obtained using an MDEFT sequence in 3-Tesla Siemens Trio scanner. These anatomical MR images were not used in source localisation and served only for post-hoc verification of anatomical labels of source dipole locations.

#### Data analysis

MEG data were analysed using the Brain Electrical Source Analysis v. 5.2.4. program (BESA, Megis, GmbH, Germany). MEG signals were visually inspected to identify head motion artefacts. Electrocardiographic and electrooculographic artefacts were removed by identifying electrocardographic and electrooculographic patterns and averaging all occurences of these patterns in MEG data. The spatio-temporal patterns related to electrocardiographic and electrooculographic artefacts were subtracted from MEG data using principal component analysis.

Clean MEG data were cut into epochs corresponding to 0.1 s of the pre-stimulus and 2.0 s of the post-stimulus timeframe. Triggers identifying the occurences of individual laser stimuli were given order numbers so as to allow selective averaging of epochs. MEG data were averaged for all stimuli corresponding to a particular order number in all 30 sets of laser stimuli. Thus, the averaged evoked fields encompassed up to 30 stimuli in each of 10 classes of laser stimuli.

The averaged MEG waveforms were analysed using source dipole analysis in BESA program. Source reconstruction was based on a spherical head model. The size of the sphere in every subject was adjusted to the individual head shape. After source reconstruction, source dipole locations were transformed into Talairach space. To transform the source locations coordinates into Talairach coordinates, the most anterior and posterior points of the sphere, the most left and right points of the sphere, as well as the most superior and inferior points of the sphere were coregistered with the normalised brain, thereby transforming the sphere into an ellipsoid approximating the shape of human brain. Data were filtered from 0.3 to 7 Hz prior to source dipole analysis. These filter settings were chosen to detect the long-latency field changes associated with cortical responses to warming of the skin [27], [28], [29], [30]. The source model was constructed using sequential strategy regarding global field power and residual variance [61], [62]. The anatomical locations of the sources in select subjects were checked by coregistering the source dipole locations, individual head shapes and MR images in BrainVoyagerQX v. 1.9 (BrainInnovation, Maastricht).

### Experiment 2

To evaluate the changes in skin temperature occurring during repeated laser stimulation and specifically to exclude the possibility that laser stimulation produced an increase of temperature reaching the threshold of stimulation of mechano-heat C nociceptors in the range of 39–42°C [20], [21], [22], the skin temperature changes occurring after each laser stimulus was recorded outside the MEG scanner. Six healthy subjects (2 females, 4 males, age 23.9±3.0 years, mean ± SD) took part in this experiment. The procedure of the experiment was identical with Experiment 1 except for the subject sitting in a chair with his/her hand resting on the desk. The infrared thermometer Fluke 80 T-IR (Fluke, Germany) was directed at 70° angle onto the stimulated area of the skin. The infrared thermometer was connected to a Thurlby 1503 digital multimeter (Thurlby Electronics, LTD., England). Maximal skin temperature in the 2.5 s interval following each laser stimulus was recorded with a 0.1°C accuracy. One temperature reading was also taken prior to the first laser stimulus in each of 30 trials; these values were used to quantify the long-term effects of repeated laser stimulation on baseline skin temperature.

### Experiment 3

To validate that repeated laser stimuli activated the C fibre system, reaction time elapsing between onset of laser stimulus and onset of the first warming or heating sensation was recorded on each laser stimulus in six subjects (1 female, 5 males, age 24.5±3.0 years, mean ± SD). Subjects were asked to press the button with their left hand as soon as they noticed a rise in skin temperature for each stimulus interval starting with presentation of the stimulus order number on the computer screen. As the button press was used for the purpose of reaction time measurement, subjects were instructed to withdraw their right hand from under the laser probe to avoid the next stimulus if they wished to do so. Subjects were trained to reposition their right hand before the next trial using the red diode light produced by the laser. The red circle, produced by a diode, was always centred to the skin area located 1 cm dorsal to the metacarpo-phalangeal joint of the 4th finger. Reaction time data were assembled from six sets of laser stimuli with identical stimulus parameters as used in Experiments 1 and 2.

### Statistical analysis

The source dipole moments, numerical rating scale values, reaction time data and temperature values were analysed using repeated measures ANOVA and linear and non-linear regression analysis in STATISTICA v. 6 (Statsoft, Inc., USA). A 95% confidence level was always employed.
